# Single-Row or Double-Row Fixation Technique for Full-Thickness Rotator Cuff Tears: A Meta-Analysis

**DOI:** 10.1371/journal.pone.0068515

**Published:** 2013-07-11

**Authors:** Qiang Zhang, Heng’an Ge, Jiaojiao Zhou, Chaoqun Yuan, Kai Chen, Biao Cheng

**Affiliations:** 1 Department of Orthopedics, Shanghai Tenth People’s Hospital, Tongji University School of Medicine, Shanghai, China; 2 First Clinical Medical College, Nanjing Medical University, Nanjing, China; Sapienza University of Rome, Italy

## Abstract

**Background:**

The single-row and double-row fixation techniques have been widely used for rotator cuff tears. However, whether the double-row technique produces superior clinical or anatomic outcomes is still considered controversial. This study aims to use meta-analysis to compare the clinical and anatomical outcomes between the two techniques.

**Methods:**

The Pubmed, Embase, and Cochrane library databases were searched for relevant studies published before November 1, 2012. Studies clearly reporting a comparison of the single-row and double-row techniques were selected. The Constant, ASES, and UCLA scale systems and the rotator cuff integrity rate were evaluated. The weighted mean differences and relative risks were calculated using a fixed-effects or random-effects model.

**Results:**

Eight studies were included in this meta-analysis. The weighted mean differences of the ASES (−0.84; P = 0.04; I^2^ = 0%) and UCLA (−0.75; P = 0.007; I^2^ = 0%) scales were significantly low in the single-row group for full-thickness rotator cuff tears. For tear sizes smaller than 3 cm, no significant difference was found between the groups no matter in Constant (P = 0.95; I^2^ = 0%), ASES (P = 0.77; I^2^ = 0%), or UCLA (P = 0.24; I^2^ = 13%) scales. For tear sizes larger than 3 cm, the ASES (−1.95; P = 0.001; I^2^ = 49%) and UCLA (−1.17; P = 0.006; I^2^ = 0%) scales were markedly lower in the single-row group. The integrity of the rotator cuff (0.81; P = 0.0004; I^2^ = 10%) was greater and the partial thickness retear rate (1.93; P = 0.007; I^2^ = 10%) was less in the double-row group. Full-thickness retears showed no difference between the groups (P = 0.15; I^2^ = 0%).

**Conclusion:**

The meta-analysis suggests that the double-row fixation technique increases post-operative rotator cuff integrity and improves the clinical outcomes, especially for full-thickness rotator cuff tears larger than 3 cm. For tear sizes smaller than 3 cm, there was no difference in the clinical outcomes between the two techniques.

**Level of Evidence:**

Level I.

## Introduction

Rotator cuff tears are one of the most common disorders of the shoulder, and they have a significant effect on daily life as a result of the loss of motion and strength. Approximately 17% to 50% of adults older than 60 years and 80% of adults older than 80 years may have rotator cuff pathologies [1]–[3].

Several techniques have been introduced over the past decade for the arthroscopic repair of rotator cuff tears. Single-row and double-row rotator cuff repairs with suture anchors are the most commonly used procedures, with both having favorable outcomes. Previous studies have reported retears and incomplete tendon healing after arthroscopic rotator cuff repair using a single-row technique despite the good clinical results [4]–[6]. The double-row technique was performed to increase the tendon-bone contact area and improve the postoperative cuff integrity [7]–[10]. However, controversy continues regarding the two techniques in the literature as few articles support the superior clinical outcomes of double-row fixation compared with single-row fixation [8], [10]–[20]. Additionally, few studies have investigated the clinical or imaging outcomes according to tear size.

The purpose of this study was to conduct a meta-analysis to compare the clinical and imaging outcomes between the single-row and double-row techniques. Our hypothesis was that double-row repairs would result in better clinical outcomes and more intact rotator cuff tendons than single-row repairs.

## Methods

The Pubmed, Cochrane library, and Embase databases were searched independently by 2 investigators (Q.Z. and H.A.G.) to retrieve relevant studies published before November 1, 2012. The search criteria “rotator cuff”, “single row”, and “double row” were used in text word searches. The “related articles” function was used to broaden the search. The reference lists of the selected articles were also manually examined to find relevant studies that were not discovered during the database searches. The corresponding authors were contacted when additional information was needed.

### Inclusion Criteria

1. Language: English
2. Prospective studies with Level I or II evidence
3. Arthroscopic repair
4. Includes both double- and single-row repairs
5. Greater than a 24-month minimum follow-up
6. Follow-up examination presenting at least one of the following outcome measurements: ASES score, Constant score, UCLA scale, and radiographic follow-up of the repaired rotator cuffs

### Exclusion Criteria

1. Retrospective study
2. Level III or IV evidence
3. Less than a 24-month minimum follow-up
4. Only report either single- or double-row repairs
5. Did not report the standard differentiation of all data
6. Included open or mini-open procedures

### Data Extraction

Data extraction of all variables and outcomes of interest and assessment of the methodological quality were performed independently by 2 readers (Q.Z. and H.A.G.). Disagreements were resolved through discussion and consensus. The methodological quality of the trials was assessed using the Cochrane Handbook for Systematic Reviews of Interventions 5.1.

### Outcomes

Both subjective and objective functional outcome measurements were used to evaluate the data. The Constant scale, American Shoulder and Elbow Surgeons scale (ASES), and University of California at Los Angeles scale (UCLA) were analyzed to determine the functional outcome. If the studies reported several functional outcome scores at different follow-up visits, the score after 24 months post-operatively was used for the study. Additionally, the radiographic follow-up of repaired rotator cuffs was compared between the groups. Rotator cuff integrity was divided into three degrees: (1) full-thickness retear, (2) partial-thickness retear and (3) integrity cuff.

### Statistical Analysis

The statistical analysis was performed using Review Manager 5.1 (Cochrane Collaboration, Nordic Cochrane Centre, Copenhagen, Denmark). Continuous variables were analyzed using the weighted mean difference, whereas categorical dichotomous variables were assessed using relative risks (RRs). P values <0.05 were considered statically significant, and the 95% confidence intervals (CIs) were reported. Homogeneity was tested by the Q statistic (significance level at P<0.10) and the I^2^ statistic (significance level at I^2^>50%). A random-effects model was used if the Q or I^2^ statistic was significant; otherwise, a fixed-effects model was used. The presence of publication bias was assessed by a visual inspection of a funnel plot and the Begg and Egger tests (with P<0.05 considered statistically significant). We also conducted subgroup analyses stratified by the size of the tear to assess the impact of tear size on the outcomes.

## Results

### Literature Search

**Figure 1:**
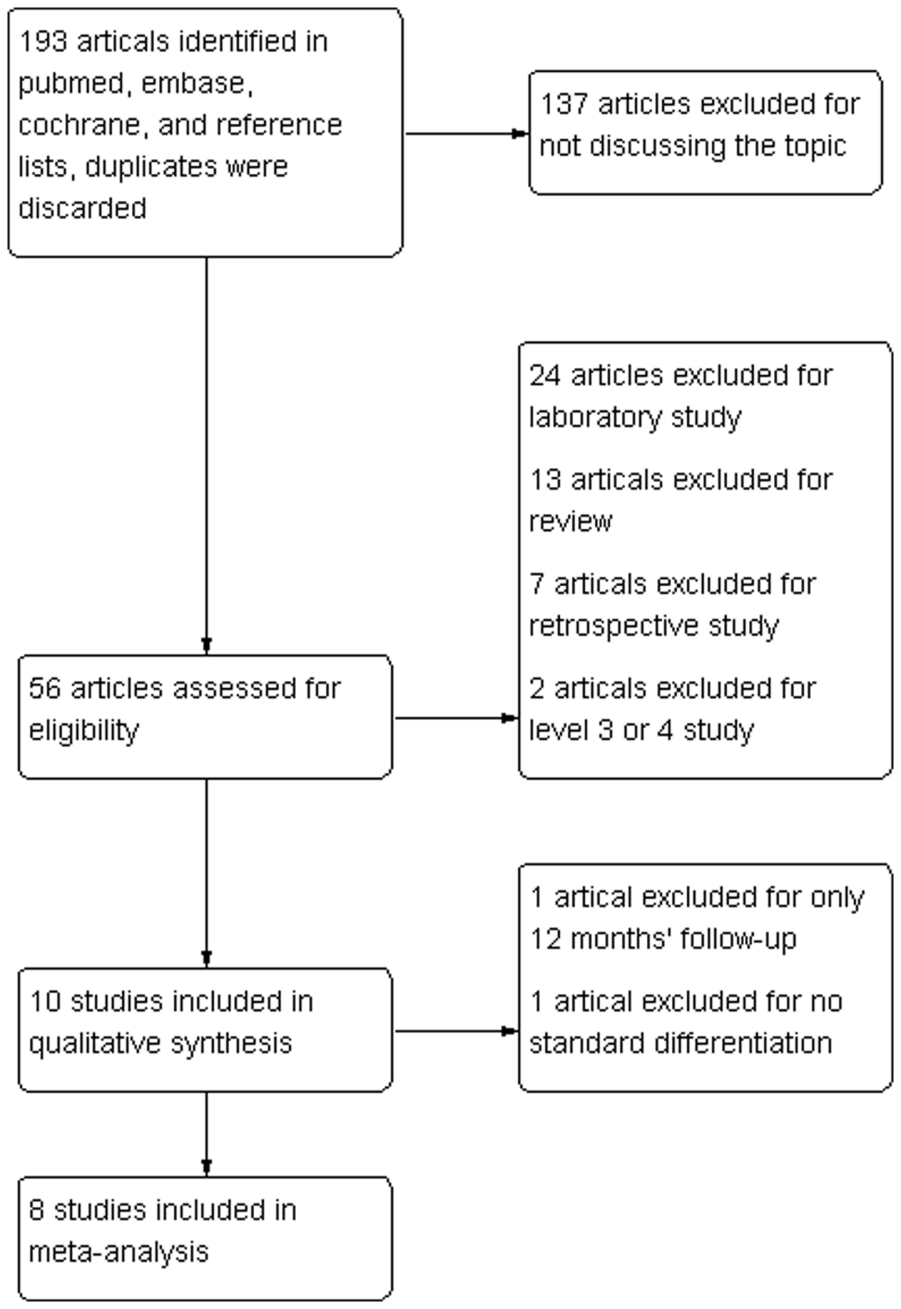
Search strategy flow diagram.

The initial literature search retrieved 193 relevant articles (duplicates were discarded). After a careful screen of the titles, 137 articles were excluded for not investigating the topic of interest. After reviewing the abstracts, 46 more articles were excluded (24 laboratory studies, 7 retrospective studies, 2 level 3 evidence studies, and 13 reviews), leaving 10 studies for further full publication review. One study was excluded because it only performed follow-up for 12 months [12]. Another study was excluded because the article did not report the standard differentiation of the data [11]. Therefore, 8 studies matched the selection criteria and were suitable for meta-analysis [8], [10], [13]–[15], [17], [21], [22]; 6 were prospective randomized control trials, and 2 were prospective cohort studies [13], [17] (Figure 1). A total of 619 patients (311 single row and 308 double row) were enrolled in the studies. The key characteristics of the included studies are summarized in Table 1. All the studies involved patients with reparable full-thickness rotator cuff tears and follow-up for at least 24 months. Among the included studies, 4 studies investigated the Constant scale, 5 studies investigated the ASES scale, 3 studies investigated the UCLA scale, and 6 studies reported post-operative imaging outcomes. Additionally, 3 studies performed subgroup analysis according to the tear size (smaller or larger than 3 cm). On review of the data extraction, there was 100% agreement between the 2 reviewers.

**Table 1:** Characteristics of the included studies.

Study	Country	Study Design	Tear Size	FinalSample Size	Age	Follow-up	Matching Outcome Measures
Peter L.C. Lapner 2012	Canada	RCT	reparable full-thickness tears of any size	39 vs. 37	56±8.9 vs. 57.8±7	24 vs. 24 months	Constant ASES Imaging
Ignacio Carbonel 2012	Spain	RCT	reparable full-thickness tears (10–50 mm)	80 vs. 80	55.79±6.3 vs. 55.21±5.0	24 vs. 24 months	Constant ASES UCLA Imaging
Hsiao-Li Ma 2012	Taiwan,China	RCT	reparable full-thickness tears larger than 10 mm	27 vs. 26	60.8 vs. 61.6	33.3 vs. 33.5 months	ASES UCLA Imaging
Kyoung Hwan Koh 2011	Korea	RCT	reparable full-thickness rotator cuff tears (2–4 cm)	31 vs. 31	61.6±8.8 vs. 61.1±9.1	31.0 vs. 32.8 months	Constant ASES UCLA Imaging
Andrea Grasso 2009	Italy	RCT	reparable full-thickness tears	37 vs. 35	58.3±10.3 vs. 55.2±6.5	24.8 _ 1.4 months	Constant
Jin-Young Park 2008	Korea	prospective cohort study	reparable full-thickness tears	40 vs. 38	57 vs. 54.4	25.1 months	Constant ASES
Francesco Franceschi 2007	England	RCT	reparable full-thickness tears	26 vs. 26	63.5 vs. 59.6	24 vs. 24 months	Imaging
Christophe Charousset 2007	France	prospective cohort study	reparable full-thickness tears	31 vs. 35	60 vs. 58	28.7 vs. 27.6 months	Imaging

**Figure 2:**
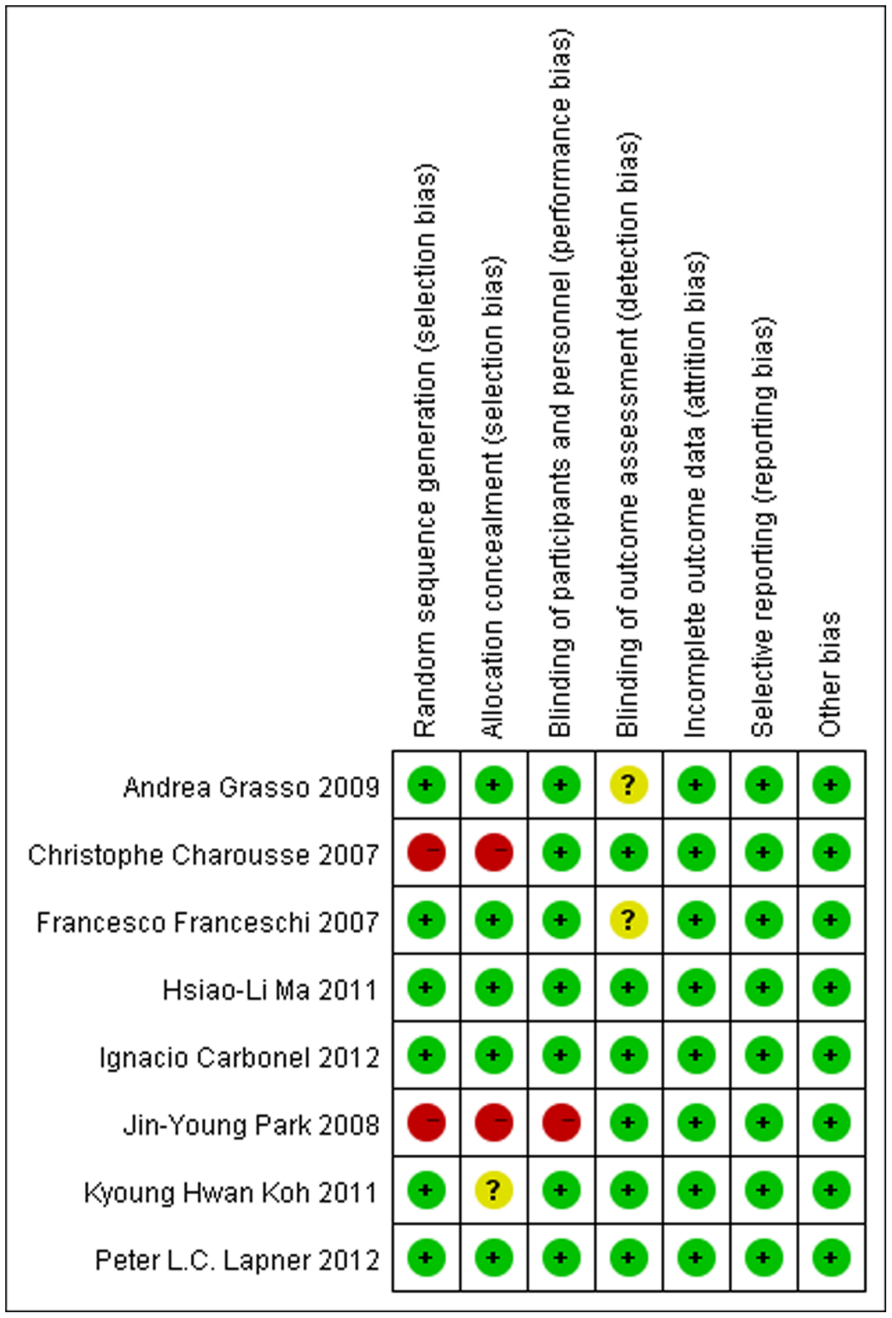
The methodological quality of the included studies.

Figure 2 summarizes the methodological quality of the studies. Most of the studies were RCTs with a high level of methodological quality. Only 2 studies were prospective cohort studies, and they also had high methodological quality despite being nonrandomized. Thus, the methodological bias of this study was low.

### Main Analysis

**Figure 3:**
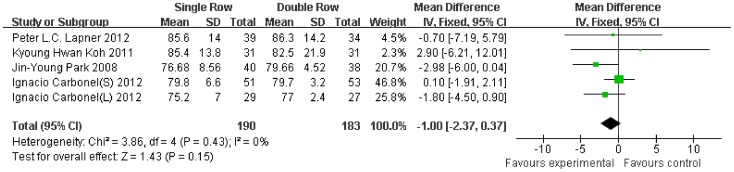
Difference of the Constant scale.

**Figure 4:**
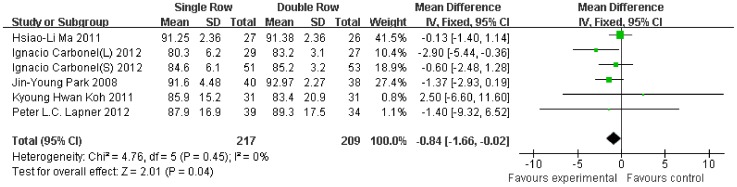
Difference of the ASES scale.

**Figure 5:**
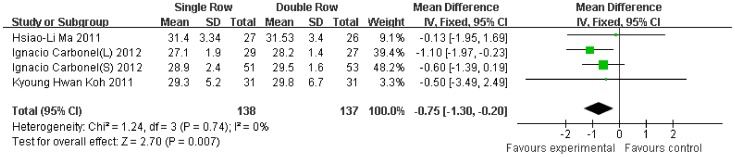
Difference of the UCLA scale.

Compared with the double-row group, the Constant scale was 1.00-fold lower (95% CI, −2.37 to 0.37; P = 0.15) (Figure 3), the ASES scale was 0.84-fold lower (95% CI, −1.66 to −0.02; P = 0.04) (Figure 4), and the UCLA scale was 0.75-fold lower (95% CI, −1.30 to −0.20; P = 0.007) (Figure 5) in the single-row group. No significant heterogeneity was found among the studies (Constant, P = 0.43, I^2^ = 0%; ASES, P = 0.45, I^2^ = 0%; UCLA, P = 0.74, I^2^ = 0%).

**Figure 6:**
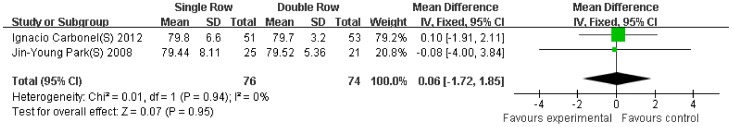
Difference of the Constant scale for small tear size.

**Figure 7:**
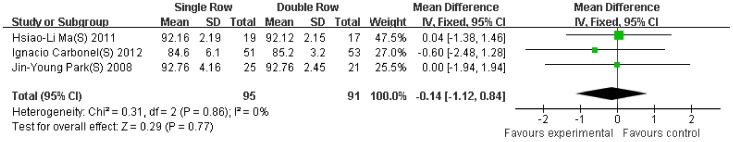
Difference of the ASES scale for small tear size.

**Figure 8:**
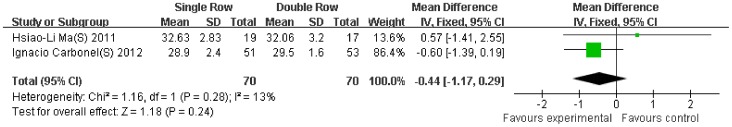
Difference of the UCLA scale for small tear size.

**Figure 9:**
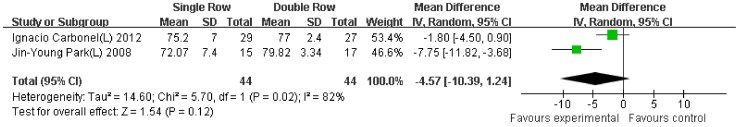
Difference of the Constant scale for large tear size.

**Figure 10:**
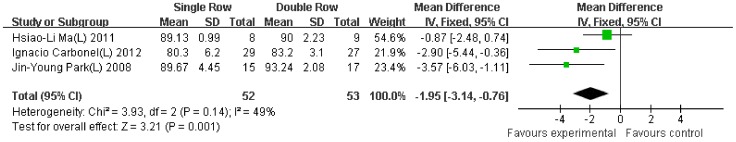
Difference of the ASES scale for large tear size.

**Figure 11:**
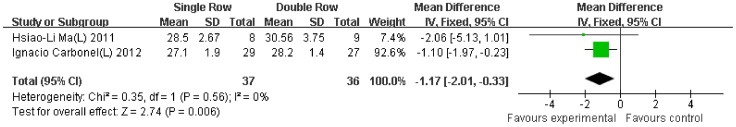
Difference of the UCLA scale for large tear size.

A subgroup analysis according to tear size was also performed. With regard to small size rotator cuff tears (<3 cm), no significant difference and heterogeneity were found between the groups for the Constant (Figure 6), ASES (Figure 7), and UCLA (Figure 8) scales. With regard to large size rotator cuff tears (>3 cm), the Constant scale (95% SI, −10.39 to 1.24; P = 0.12) (Figure 9) showed no difference between the groups, but the ASES (P = 0.001) (Figure 10) and UCLA (P = 0.006) (Figure 11) scales were markedly lower in the single-row group. Because significant heterogeneity was observed for the Constant scale (P = 0.02, I^2^ = 82%), the random-effects model was then used as no significant clinical heterogeneity was found between the studies.

**Figure 12:**
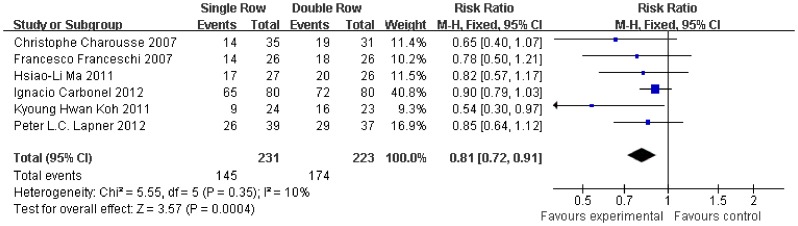
Risk of rotator cuff integrity.

**Figure 13:**
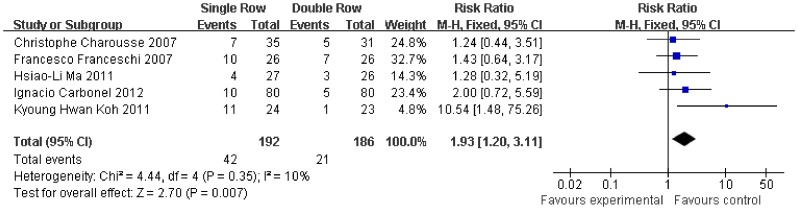
Risk of partial thickness retear of the rotator cuff.

**Figure 14:**
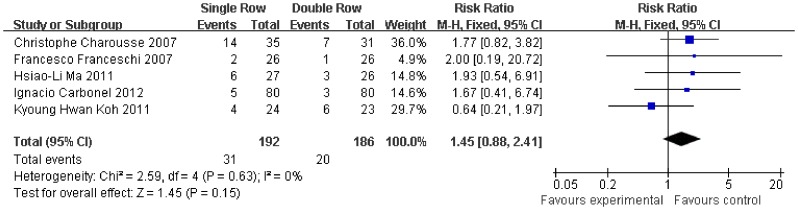
Risk of full-thickness retear of the rotator cuff.

With respect to post-operative rotator cuff integrity, the RR of integrity cuff was 20% lower in the single-row group (RR, 0.81, 95% CI, 0.72 to 0.91; P = 0.0004) (Figure 12). The RR of partial-thickness retears was 93% higher in the single-row group (RR, 1.93, 95% CI, 1.20 to 3.11; P = 0.007) (Figure 13). In contrast, no significant difference was observed for full-thickness retears (RR, 1.45, 95% CI, 0.88 to 2.41; P = 0.15) (Figure 14). No significant heterogeneity was found among these studies.

### Publication Bias

Funnel plots demonstrated no visual evidence of publication bias.

## Discussion

The treatment of rotator cuff tears has progressed rapidly over the last few years. New techniques such as arthroscopy, improved fixation devices, and several fixation methods have made it possible to release pain and improve the function of the patients. Single-row and double-row techniques were mostly used, with both having favorable results. The double-row fixation procedure is more complex and more expensive. However, it can restore the rotator cuff footprint and produce better clinical outcomes. However, few studies exist showing better functional and radiological outcomes with the use of the double-row technique. As a result, the cost-effectiveness of the double-row technique has been questioned recently.

With the present meta-analysis of level 1 and 2 evidence prospective control studies, we were able to support our primary hypothesis that double-row rotator cuff repair would lead to better clinical and radiographic results compared with single-row repair, especially for those tears larger than 3 cm.

The clinical scale systems were the most commonly used and most effective methods to evaluate the surgery. Although previous studies showed higher healing rates, no studies have reported better clinical outcomes. Thus, we could not conclude that the double-row technique was better than the single row. In this study, regardless of the tear size, there was a significantly higher score in the double-row group for the ASES and UCLA scales but no significant difference for the Constant scale. Although the 95% confidence intervals of the postoperative mean difference of the Constant scale contained zero, we could not ignore that the low P value and the overall trend of the 95% CI to less than zero. The patient number may not have been large enough to detect the difference.

The subgroup analysis for the three scale systems showed that when the tear size was smaller than 3 cm, there was no significant difference between the two procedures. However, for tears larger than 3 cm, the scores were markedly higher in the double-row group for the ASES and UCLA scales. Because there was significant heterogeneity for the Constant scale, a random-effects model was used to evaluate the difference. However, no difference was found for the Constant scale (95% CI, −10.39 to 1.24; P = 0.12), although there was a trend towards a significant difference. Additionally, for the Constant and UCLA scales comparing large size tears, only two studies were enrolled for both. As a result, the evidence for the Constant and UCLA scales was not enough to support this conclusion. Therefore, the ASES scale (3 enrolled studies), which suggested superior outcomes for the double-row technique with a high level of evidence, is important.

Given the evidence above, the double-row fixation technique showed a trend towards superior functional outcomes regardless of the tear size. Additionally, for tear sizes smaller than 3 cm, the two procedures produced equal effects. However, for tear sizes larger than 3 cm, the double-row technique was much better.

The post-operative imaging outcomes also showed that the double-row procedure resulted in more integrity, fewer partial thickness retears, and an equal number of full-thickness retears. As a result, we can conclude that anatomic healing was significantly superior with the double-row fixation method. However, among the 6 studies comparing rotator cuff integrity, only one performed subgroup analysis according to tear size [22]. Thus, we cannot determine if the double-row technique is also superior for small size rotator cuff tears.

Some limitations exist in this meta-analysis. First, there were no subgroups when comparing the rotator cuff integrity. Whether the small size rotator cuff tears showed differences between the groups for rotator cuff integrity remains unknown. Second, there are many other double-row techniques, such as the transosseous equivalent [23], which is reported to produce a better footprint and functional outcomes than simple double-row fixation. This study did not distinguish among the different double-row fixation techniques. The transosseous equivalent technique may be able to produce even better outcomes than the single low technique. Third, for the subgroup analysis of the Constant and UCLA scales, only two studies were used, which may result in bias. More studies were needed. Fourth, it was unable to perform a meta-analysis in regarding with the harms like complications. However, there was no evidence that showed any difference between the two procedures for post-operative complications. Lastly, this study did not investigate the strength and range of motion post-operatively, which are more objective outcomes than the scale system.

Although this meta-analysis showed many limitations, it still has a high level of evidence and may be used to guide future clinical work.

### Conclusion

In conclusion, this meta-analysis suggests that the double-row fixation technique increases the post-operative rotator cuff integrity and improves the clinical outcomes, especially for tear sizes greater than 3 cm. For tear sizes less than 3 cm, there was no difference in clinical outcomes between the two techniques.

## Supporting Information

Table S1 Completed PRISMA checklist. Table S1 presents the completed PRISMA checklist for the meta-analysis.
(DOC)

## References

[1] Milgrom C, Schaffler M, Gilbert S, van Holsbeeck M (1995) Rotator-cuff changes in asymptomatic adults. The effect of age, hand dominance and gender. J Bone Joint Surg Br 77: 296–298. 7706351

[2] Tempelhof S, Rupp S, Seil R (1999) Age-related prevalence of rotator cuff tears in asymptomatic shoulders. J Shoulder Elbow Surg 8: 296–299. 10471998 10.1016/s1058-2746(99)90148-9

[3] Lehman C, Cuomo F, Kummer FJ, Zuckerman JD (1995) The incidence of full thickness rotator cuff tears in a large cadaveric population. Bull Hosp Jt Dis 54: 30–31. 8541777

[4] Boileau P, Brassart N, Watkinson DJ, Carles M, Hatzidakis AM, et al (2005) Arthroscopic repair of full-thickness tears of the supraspinatus: does the tendon really heal? J Bone Joint Surg Am 87: 1229–1240. 15930531 10.2106/JBJS.D.02035

[5] Charousset C, Duranthon LD, Grimberg J, Bellaiche L (2006) [Arthro-C-scan analysis of rotator cuff tears healing after arthroscopic repair: analysis of predictive factors in a consecutive series of 167 arthroscopic repairs]. Rev Chir Orthop Reparatrice Appar Mot 92: 223–233. 16910604 10.1016/s0035-1040(06)75729-4

[6] Gerber C, Fuchs B, Hodler J (2000) The results of repair of massive tears of the rotator cuff. J Bone Joint Surg Am 82: 505–515. 10761941 10.2106/00004623-200004000-00006

[7] Galatz LM, Ball CM, Teefey SA, Middleton WD, Yamaguchi K (2004) The outcome and repair integrity of completely arthroscopically repaired large and massive rotator cuff tears. J Bone Joint Surg Am 86-A: 219–224. 14960664 10.2106/00004623-200402000-00002

[8] Koh KH, Kang KC, Lim TK, Shon MS, Yoo JC (2011) Prospective randomized clinical trial of single- versus double-row suture anchor repair in 2- to 4-cm rotator cuff tears: clinical and magnetic resonance imaging results. Arthroscopy 27: 453–462. 21444007 10.1016/j.arthro.2010.11.059

[9] Smith CD, Alexander S, Hill AM, Huijsmans PE, Bull AM, et al (2006) A biomechanical comparison of single and double-row fixation in arthroscopic rotator cuff repair. J Bone Joint Surg Am 88: 2425–2431. 17079400 10.2106/JBJS.E.00697

[10] Lapner PL, Sabri E, Rakhra K, McRae S, Leiter J, et al (2012) A multicenter randomized controlled trial comparing single-row with double-row fixation in arthroscopic rotator cuff repair. J Bone Joint Surg Am 94: 1249–1257. 22810395 10.2106/JBJS.K.00999

[11] Aydin N, Kocaoglu B, Guven O (2010) Single-row versus double-row arthroscopic rotator cuff repair in small- to medium-sized tears. J Shoulder Elbow Surg 19: 722–725. 20303287 10.1016/j.jse.2009.11.053

[12] Burks RT, Crim J, Brown N, Fink B, Greis PE (2009) A prospective randomized clinical trial comparing arthroscopic single- and double-row rotator cuff repair: magnetic resonance imaging and early clinical evaluation. Am J Sports Med 37: 674–682. 19204365 10.1177/0363546508328115

[13] Charousset C, Grimberg J, Duranthon LD, Bellaiche L, Petrover D (2007) Can a double-row anchorage technique improve tendon healing in arthroscopic rotator cuff repair?: A prospective, nonrandomized, comparative study of double-row and single-row anchorage techniques with computed tomographic arthrography tendon healing assessment. Am J Sports Med 35: 1247–1253. 17452513 10.1177/0363546507301661

[14] Franceschi F, Ruzzini L, Longo UG, Martina FM, Zobel BB, et al (2007) Equivalent clinical results of arthroscopic single-row and double-row suture anchor repair for rotator cuff tears: a randomized controlled trial. Am J Sports Med 35: 1254–1260. 17554104 10.1177/0363546507302218

[15] Grasso A, Milano G, Salvatore M, Falcone G, Deriu L, et al (2009) Single-row versus double-row arthroscopic rotator cuff repair: a prospective randomized clinical study. Arthroscopy 25: 4–12. 19111212 10.1016/j.arthro.2008.09.018

[16] Mihata T, Watanabe C, Fukunishi K, Ohue M, Tsujimura T, et al (2011) Functional and structural outcomes of single-row versus double-row versus combined double-row and suture-bridge repair for rotator cuff tears. Am J Sports Med 39: 2091–2098. 21785001 10.1177/0363546511415660

[17] Park JY, Lhee SH, Choi JH, Park HK, Yu JW, et al (2008) Comparison of the clinical outcomes of single- and double-row repairs in rotator cuff tears. Am J Sports Med 36: 1310–1316. 18413680 10.1177/0363546508315039

[18] Pauly S, Gerhardt C, Chen J, Scheibel M (2010) Single versus double-row repair of the rotator cuff: does double-row repair with improved anatomical and biomechanical characteristics lead to better clinical outcome? Knee Surg Sports Traumatol Arthrosc 18: 1718–1729. 20737134 10.1007/s00167-010-1245-7

[19] Pennington WT, Gibbons DJ, Bartz BA, Dodd M, Daun J, et al (2010) Comparative analysis of single-row versus double-row repair of rotator cuff tears. Arthroscopy 26: 1419–1426. 20875720 10.1016/j.arthro.2010.03.013

[20] Gerhardt C, Hug K, Pauly S, Marnitz T, Scheibel M (2012) Arthroscopic Single-Row Modified Mason-Allen Repair Versus Double-Row Suture Bridge Reconstruction for Supraspinatus Tendon Tears: A Matched-Pair Analysis. Am J Sports Med. 10.1177/0363546512462123 23104608

[21] Carbonel I, Martinez AA, Calvo A, Ripalda J, Herrera A (2012) Single-row versus double-row arthroscopic repair in the treatment of rotator cuff tears: a prospective randomized clinical study. Int Orthop 36: 1877–1883. 22584619 10.1007/s00264-012-1559-9 PMC3427450

[22] Ma HL, Chiang ER, Wu HT, Hung SC, Wang ST, et al (2012) Clinical outcome and imaging of arthroscopic single-row and double-row rotator cuff repair: a prospective randomized trial. Arthroscopy 28: 16–24. 21982391 10.1016/j.arthro.2011.07.003

[23] Toussaint B, Schnaser E, Bosley J, Lefebvre Y, Gobezie R (2011) Early structural and functional outcomes for arthroscopic double-row transosseous-equivalent rotator cuff repair. Am J Sports Med 39: 1217–1225. 21427446 10.1177/0363546510397725

